# Assessment of health service delivery capacities, health providers’ knowledge and practices related to type 2 diabetes care in Kinshasa primary healthcare network facilities, Democratic Republic of the Congo

**DOI:** 10.1186/s12913-015-0679-5

**Published:** 2015-01-22

**Authors:** Remy Y Kapongo, Aimée M Lulebo, Eric M Mafuta, Paulin B Mutombo, Jean Claude M Dimbelolo, Isidore E Bieleli

**Affiliations:** Internal Medicine Service, Friendship Sino-Congolese hospital, Kinshasa, Democratic Republic of Congo; Department of Internal Medicine, University Clinics of Kinshasa, Faculty of Medicine, University of Kinshasa, Kinshasa, Democratic Republic of Congo; Kinshasa School of Public Health, School Public Health, Faculty of Medicine, University of Kinshasa, Po Box 11850, Kinshasa 1, DR Congo; Diabetes Education Center, Kinshasa, Democratic Republic of Congo

**Keywords:** Type 2 diabetes, Primary healthcare, Guidelines, Sub-Saharan Africa, Democratic Republic of the Congo, Service delivery, LMIC

## Abstract

**Background:**

Democratic Republic of the Congo (DRC) is experiencing an increase in the morbi-mortality related to Non Communicable Diseases (NCD). The reform of DRC health system, based on Health District model, is needed in order to tackle this public issue. This article used 2006 International Diabetes Federation (IDF)’s guidelines to assess the capacities of health facilities belonging to Kinshasa Primary Health Care Network (KPHCN) in terms of equipments, as well as the knowledge, and the practice of their health providers related to type 2 diabetes care.

**Methods:**

A multicentric cross-sectional study was carried in 18 Health Facilities (HF) of KPHCN in charge of the follow-up of diabetic patients. The presence of IDF recommended materials and equipment was checked and 28 health providers were interviewed about their theoretical knowledge about patients’ management and therapeutic objectives during recommended visits. Chi square test or Fisher exact test was used to compare proportions and the Student *t*-test to compare means.

**Results:**

The integration of NCD healthcare in the KPHC network is feasible. The majority of HF possessed IDF recommended materials except for the clinical practice guidelines, urinary test strips, and monofilament, available in only one, two and four HF, respectively. KPHCN referral facilities had required materials for biochemical analyses, the ECG and for the fundus oculi test. Patients’ management is characterized by a lack of attention on the impairment of renal function during the first visits and a poor respect of recommended practices during quarterly and annual visits. A poor knowledge of the reduction of cardiovascular risk factors-related therapeutic objectives has been also reported.

**Conclusion:**

The capacities, knowledge, and practice of T2D care were poor among HF of KPHCN. The lack of equipment and training of healthcare professionals should be supplied even to those who are not medical doctors. Special attention must to be put on the clinical practice guidelines formulation and sensitization and on supervision.

## Background

Type 2 diabetes is one of the main non-communicable diseases (NCD) and is also an important cardiovascular risk factor. The World Health Organization (WHO) projects that the total number of diabetics will increase, worldwide, to more than 300 million by 2025 with at least two thirds of them living in developing countries [1]. Sub-Saharan Africa countries (SSA) amongst which the Democratic Republic of the Congo (DRC) are experiencing an increase in the morbi-mortality related to non-communicable diseases. These countries counted 7 million diabetics in 2000 and are projected to have more than 15 million in 2030 [2]. They are experiencing one of the most rapid epidemiological transitions and are thence confronted with a double morbid burden in a context where their health systems are essentially oriented to manage infectious diseases [3-9]. Then non-communicable diseases remain globally neglected in SSA countries and most countries have not positioned NCD in their agenda yet [10].

Therefore, it was vital that DRC as other SSA countries reforms and updates its health system to this new epidemiological situation [8-14]. In DRC, the health system reform was promoted through the integration of non-communicable diseases care management at primary health level in the health policy but the implementation of this policy was not effective everywhere in the country [15-17]. Thus, to explore the feasibility of the integration of NCD in primary health level package, a pilot experience was implemented in some health facilities of Kinshasa, capital city of DRC since 1980’s. These health facilities were included in a network comprising some health centers around the referral healthcare facilities was called Kinshasa primary healthcare (KPHC) network. These facilities have agreed to open a special unit for diabetes care provision and have, for that, agreed to provide their nurses with an in-service training regarding diabetes care. The care delivery for T2D care in KPHC network is organized in two levels connecting the health centers to the referral hospital. In this network primary level facilities are supervised by healthcare providers from the referral level. They have worked for more than 3 decades with an integrated approach in order to improve diabetes care. Currently, this network comprises about 51 structures and allows a follow-up of the diabetics living in Kinshasa. At the actual state of existing data, other health centers outside this network have not already integrated diabetes care management. However, current studies carried out in DRC showed that the referral healthcare facilities attached to this network still continue to receive patients presenting severe complications of diabetes [18-21]. This situation raises the question of the effectiveness of type 2 diabetes care management integration in primary healthcare level through this network particularly in terms of health service delivery capacities, and health providers’ knowledge and practices related to type 2 diabetes care.

The International Diabetes Federation (IDF) has developed clinical practice guidelines of type 2 diabetes care in SSA [22] that contain instructions for a better clinical practice in order to improve type 2 diabetes care in countries with limited resources. This article used these IDF guidelines as reference to assess the capacities of health facilities belonging to this network in terms of equipments, as well as the knowledge, and the practice of their health providers related to type 2 diabetes care.

## Methods

A multicentric cross-sectional study was carried out in Kinshasa primary diabetes healthcare network facilities from January to march 2010. Eighteen health facilities representing one–third of these 51 facilities were systematically sampled. Two of the three referral facilities of this network were also studied. Data were collected through face-to-face interview and observation using respectively a questionnaire and a check-list both developed with reference to 2006 IDF guidelines [22]. All research tools were pretested.

All healthcare providers responsible for care in the selected healthcare facilities, medical supervisors, medical doctors and nurses, were interviewed. The presence of materials and equipment recommended by the IDF clinical practice guidelines were observed and marked on a check-list [22]. The evaluation of the capacities of the healthcare facilities concerned, at the first or health centers level, human resources, their types (head nurse, nurse, educator of the diabetics for both levels) and numbers. At the secondary or reference health centers level, we looked for the presence of a technician of laboratory, a doctor supervisor, a chiropodist and a dietitian. The evaluation also concerned the presence of equipment and materials according to the guidelines of IDF. These guidelines include the urinary strips, the glucometer, the sphygmomanometer, the balance scales, the height gauge, the measuring tape and the monofilament. For the secondary level, we focused more on the availability of diapason, of reflex hammer, of the materials for the fundus oculi, the electrocardiogram (ECG), the plasmatic level of glucose, lipid, creatinine and glycated hemoglobin (HbA1c).

The evaluation of healthcare providers concerned their theoretical knowledge of action to take in front of a diabetic patient during initial, quarterly, and annual visits; the knowledge of the objectives regarding the control of blood pressure (BP), glycemia, HbA1c, the impairment of renal function, smoking, obesity and dyslipidemia were pursued. The collected information was reported on a data collection form.

### Statistical analysis

The collected data were entered in Epi Info 6.03 software and exported for analyses to SPSS 12.0. The quantitative data were presented in the form of average ± standard deviation and the qualitative data in the form of proportions (%). The comparison of proportions was realized by means of Chi square test or Fisher exact test and that of the averages by Student *t*-test. A p value < 0.05 was considered as statistically significant. The study protocol was submitted to and approved by the Ethics Committee of the School of Public health of the University of Kinshasa. All participants gave their written informed consent according to the Helsinki declaration II.

## Results

Twenty healthcare facilities and twenty-eight healthcare providers of the Kinshasa primary healthcare network were studied (0% of non-responders). These facilities have integrated diabetes care in their package. Eighteen primary level health structures were daily under the responsibility of a head nurse of A2 (40%) and A3 (35%) levels. All persons in charge of diabetics’ care asserted having benefited from the in-service job training about type 2 diabetes care (100%).

The majority of the evaluated health facilities was provided with the materials recommended by the IDF except for the clinical practice guidelines that were available only in a single facility, urinary test strips found in 2 facilities and monofilament available in 4 facilities. Both studied secondary health facilities were provided with materials required for biochemical analyses, the ECG and for the fundus oculi test. However, none of these structures possessed a diapason or a reflex hammer for the neurological exploration and the test to assess HbA1c level.

Concerning the human resources, the health facility had on average 2 nurses, 3 community relays associated with the activities of diabetes, a technician of laboratory, a person in charge of the education of diabetics. The secondary level health structures had in addition, medical doctors. However none of these structures possessed a chiropodist or a dietitian.

Healthcare providers had on average 15.17 ± 8.71 years of experience; this was relatively identical for both levels of care (p = 0.079). The primary level healthcare structures benefited from the supervision of medical doctors and nurses from the secondary level. The average number of health centers supervised by medical doctors (4.33 ± 1.96) was greater than that of those supervised by nurses (3.66 ± 0.57) (p < 0.001).

Concerning the practices by the healthcare providers during their various visits, more than 6/10 of healthcare providers declared to proceed, in the first visit, to the anamnesis, to the measurement of PA and height, to the dosage of glycemia and to not only treatment but also to education of patients. Nevertheless, less than 1/4 of healthcare providers paid attention to the extension record of diabetes complications. Healthcare providers of the secondary level were more effective than those of the primary level regarding the determination of the body mass index (BMI) (p = 0.013), the realization of the fundus oculi (p = 0.027) and the dosage of blood lipids (p = 0.05) (Table 1).

**Table 1:** **Proportion of healthcare providers who took IDF first visit recommended actions**

**Recommended actions**	**Total n(%)**	**Primary level n(%)**	**Secondary level n(%)**	**p**
Anamnesis	16(57.1)	13(54.8)	3(75)	0.436
Blood Pressure	22(78.6)	18(75)	4(100)	0.259
Weight	21(75)	17(70.8)	4(100)	0.212
Height	18(64.3)	14(58.3)	4(100)	0.107
BMI	12(42.9)	8(33.3)	4(100)	0.013
Foot examination	8(28.6)	6(25)	2(50)	0.306
Fundus oculi	4(14.3)	2(8.3)	2(50)	0.027
Glycemia	25(89.3)	21(87.5)	4(100)	0.454
Lipid	6(21.4)	3(12.5)	3(75)	0.005
Creatinine	6(21.4)	5(20.8)	1(25)	0.851
HbA1c	4(14.3)	3(12.5)	1(25)	0.508
Electrocardiography	4(14.3)	3(12.5)	1(25)	0.508
Education of patients	25(89.3)	21(87.5)	4(100)	0.454
Dietary advice	21(75.0)	18(75)	3(75)	1.000
Medicines	21(75.0)	17(70.8)	4(100)	0.212

In general, the actions recommended by the IDF during the quarterly visits were especially less recommended at the primary health care level (Table 2). Less than 4/10 healthcare providers applied actions recommended for the annual visits. However, the healthcare providers of the secondary level seemed to be more regular to prescribe these actions (Table 3).

**Table 2:** **Proportion of healthcare providers who took IDF quaterly visit recommended actions**

**Recommended actions**	**Total n(%)**	**Primary level n(%)**	**Secondary level n(%)**	**p**
Anamnesis	5(17.9)	4(16.7)	1(25)	0.687
Blood pressure	8(28.6)	6(25)	2(50)	0.306
Weight	7(25)	5(20.8)	2(50)	0.212
Foot examination	9(32.1)	7(29.2)	2(50)	0.409
Glycemia	8(28.6)	5(20.8)	3(75)	0.026
Lipid	5(17.9)	3(12.5)	2(50)	0.070
HbA1c	6(21.4)	3(12.5)	3(75)	0.005
Protenuria	6(21.4)	3(12.5)	3(75)	0.005
Education of patients	8(28.6)	5(20.8)	3(75)	0.026
Dietary advices	5(17.9)	3(12.5)	2(50)	0.070
Evolution of treatment	6(21.4)	4(16.7)	2(50)	0.133

**Table 3:** **Proportion of healthcare providers who took IDF annual visit recommended actions**

**Recommended actions**	**Total n(%)**	**Primary level n(%)**	**Secondary level n(%)**
Anamnesis	6(21,4)	5(20,8)	1(25)
Blood pressure	8(28,6)	5(20,8)	3(75)
Weight	8(28,6)	5(20,8)	3(75)
Height	8(28,6)	5(20,8)	3(75)
Foot examination	7(25)	4(16,7)	3(75)
Fundus oculi	6(21,4)	3(12,5)	3(75)
Glycemia	10(35,7)	7(29,2)	3(75)
Lipid	8(28,6)	5(20,8)	3(75)
Creatinine	9(32,1)	6(25)	3(75)
HbA1c	7(25)	4(16,7)	3(75)
Electrocardiography	7(25)	4(16,7)	3(75)
Education of patients	10(35,7)	7(29,2	3(75)
Dietary advices	10(35,7)	7(29,2	3(75)
Medicines	8(28,6)	5(20,8)	3(75)

Regarding the knowledge of the therapeutic objectives, about 50% of healthcare providers had provided the objectives to achieve for the reduction of the obesity and the hypertension rates. However, less than 20% specified the objectives related to the impairment of renal function, HbA1c, lipids and glycemia levels and smoking cessation. Healthcare providers of the secondary level had a better knowledge of the objectives regarding BP (p = 0.01), glycemia (p = 0.028), smoking cessation (p = 0.031), obesity (p = 0.031), total cholesterol (p = 0.018) and the HDL-C (p = 0.05) (Table 4). The level of knowledge of therapeutic objectives was related to the level of healthcare providers training and to the level of their responsibilities.

**Table 4:** **The knowledge of healthcare providers on the therapeutic objectives (n = 28)**

**Therapeutic objectives**	**Total n(%)**	**Primary level n(%)**	**Secondary level n(%)**	**p**
Blood pressure < 130/80 mmHg	13(46.4)	10(41.7)	3(75.0)	0.010
Glycemia < 100 mg/dl	4(14.3)	2(8.3)	3(75.0)	0.028
HbA1c < à 7%	5(17.9)	3(12.5)	3(75.0)	0.139
Kidney failure : control the expansion	5(17.9)	3(12.5)	3(75.0)	0.139
Smoking : cessation	5(17.9)	10(41.7)	4(100.0)	0.031
Obesity : lose weight	14(50.0)	10(41.7)	4(100.0)	0.031
Total Cholesterol total < 200 mg/dl	2(7.1)	2(8.3)	0(0.0)	0.018
HDL-C > 50 mg/dl	3(10.7)	3(12.5)	0(0.0)	0.005
LDL-C < 150 mg/dl	3(10.7)	3(12.5)	0(0.0)	0.174
Triglycerides < 150 mg/dl	2(7.1)	2(8.3)	0(0.0)	0.181

## Discussion

The present study sought to investigate the capacities of health facilities belonging to KPHC network in terms of equipments, as well as the knowledge, and the practice of their health providers related to type 2 diabetes care. This study found that the clinical diabetes guidelines were not available in all the facilities except for one; this was also the case for the urinary test strips and monofilament. The following results were also noticed: a lack of renal function assessment during the first visits; a poor respect of the IDF quarterly and annual visits recommended practices; a poor knowledge of T2D therapeutic objectives namely body weight, blood pressure, renal function, glycemia and lipidemia control; and smoking cessation. These shortages were found more at the primary level than at the secondary level, then healthcare facilities were provided with the required materials except for those used for neurological exploration and the dosage of HbA1c. They had required staff except chiropodist and dietary advisors in the referral facilities. The integration of care for non-communicable diseases in the primary health care facilities network is feasible. These findings show that the health district system can allow the integration of other activities if the required equipment and the training of healthcare providers are supplied even to those who are not medical doctors [23-25].

The model used in DRC, comparable to that described by Coleman et al. (1998), is based on the use of therapeutic guidelines by nurses [26,27]. If well followed, these guidelines can help nurses to better control the majority of non-communicable disease cases.

The availability of clinical practice guidelines at the primary level care reported in this study has not been assessed by several previous studies carried out in low–income countries. Albert et al. [28] looking for barriers in the type 2 diabetes care at the primary level focused only on the availability of medicines. The lack of these clinical practice guidelines in healthcare facilities can be an important indicator that might help to understand the quality of the care in first level healthcare facilities [26,29].

Indeed, health centers in DRC are facilities of devolution of the hospital and supply healthcare by using therapeutic guidelines. These guidelines, even the most recent ones, do not include clearly directives for the care and the follow-up of chronic diseases such as diabetes and high blood pressure [27]. The lacks of clinical practice guidelines deprive healthcare providers, usually poorly trained [30], of an important help for patient care management. The non availability of clinical practices guidelines could probably explain the non-respect of the health action or practices to be recommended or carried out during the visits and the poor knowledge of the therapeutic objectives even if previous studies already revealed this inadequate compliance with the therapeutic guidelines at the primary level [31]. However, Renders et al. underline that if a nurse is well trained and if the detailed protocol of care to provide is available, the nurse can even assume the responsibilities usually held by medical doctors [31]. Furthermore, other studies carried out have shown that the use of clinical practice guidelines have improved either health providers’ knowledge and practices or the control of risk factors [26,30].

The clinical guidelines constitute the cornerstone of the healthcare model used in rural areas in South Africa [26]. These clinical protocols allowed nurses to effectively control several cases of non-communicable diseases [26].

The non-respect of recommendations during visits and the ignorance of therapeutic objectives help understand the quality of care provided to patients and the complications often noticed in patients referred to the secondary and tertiary level.

The lack of urinary test strips and monofilament at the primary level in charge of screening of non-communicable diseases, the follow-up and the lack or shortage of the materials needed for the neurological exploration and those required for the measurement of glycated hemoglobin at the secondary level of care constitute a barrier to the access to the diagnosis and to the treatment according to Hall et al. [12] and could explain the increased rate of non-diagnosed diabetics and the poor glycemia control [12].

The absence of chiropodist and dietary advisors at the secondary level of care may be explained by the fact that, at the secondary level in the Congolese health system, healthcare is provided by general practitioners. The latter are required to carry on the patient’s foot examination and to provide dietary recommendations. However, some hospitals especially in urban zones may be provided with nutritionists.

The lack of attention to the renal function by healthcare providers especially at the primary level has been reported by several authors among whom Whiting et al. [32]. The non- application of recommended practices during visits and the lack of knowledge of the therapeutic objectives related to diabetes care and its complications can be explained by the lack of training and the non-respect of the recommendations prescribed in the clinical guidelines.

However, this study has some limitations. The first is related to the cross-sectional nature of the study, which prevented this study from inferring causation. The second limitation is related to the small sample size which makes findings unrepresentative of the country. The third limitation is due to the fact that we targeted only healthcare facilities belonging to the network of primary healthcare facilities and did not include the lucrative private healthcare facilities that also offer care to the population of Kinshasa. This results to a selection and respondent bias. However, we think, based on our knowledge and experience of the field, this is not a major limitation since the great majority of diabetic patients seek care in these abovementioned primary healthcare facilities rather than the private ones. It is also worth noting that healthcare facilities were randomly selected based on a list drawn up with the approval of supervising facilities. Finally, there is a limitation of data collection. In this study, medical staff self reporting was used in spite of observation of practice. To the best of our knowledge, this study is the first to have focused on the delivery of healthcare service for non-communicable diseases and to have found the non adequacy of this delivery in DRC like in many other developing countries. In addition, this study can be a starting point to improve the integration of type 2 diabetes care management in DRC.

## Conclusion

In the present study, we have demonstrated that the delivery of healthcare services for non-communicable diseases was hindered by the limited skills of healthcare providers on following the recommendations for good clinical practice from the guidelines, with little knowledge of therapeutic goals. This situation was mainly due to the lack of these clinical practice guidelines and some equipments. The results of the study suggest that the improvement of the healthcare service delivery for non-communicable diseases requires training healthcare providers, writing and sensitizing clinical practice guidelines, making available essential equipments available and strengthening the supportive supervision.
